# Development of Eco-Friendly Nanomembranes of Aloe vera/PVA/ZnO for Potential Applications in Medical Devices

**DOI:** 10.3390/polym14051029

**Published:** 2022-03-04

**Authors:** Muhammad Usman Munir, Daiva Mikucioniene, Haleema Khanzada, Muhammad Qamar Khan

**Affiliations:** 1 Department of Production Engineering, Faculty of Mechanical Engineering and Design, Kaunas University of Technology, LT-51424 Kaunas, Lithuania; haleema.khanzada@ktu.edu; 2 Nanotechnology Research Lab, Department of Textile and Clothing, Faculty of Engineering and Technology, National Textile University Karachi Campus, Karachi 74900, Pakistan; drqamar@ntu.edu.pk

**Keywords:** electrospinning, nanofibers, aloe vera, zinc oxide nanoparticles, antimicrobial activity, polyvinyl alcohol

## Abstract

Due to the current COVID-19 pandemic, there is a crucial need for the development of antimicrobial and antiviral personal protective equipment such as facemasks and gowns. Therefore, in this research we fabricated electrospun nanofibers composite with polyvinyl alcohol, aloe vera, and zinc oxide nanoparticles for end application in medical devices. Electrospun nanofibers were made with varying concentrations of aloe vera (1%, 2%, 3%, 4%) having a constant concentration of ZnO (0.5%) with varying concentrations of ZnO nanoparticles (1%, 2%, 3%, 4%) having a constant concentration of aloe vera (0.5%). To check the morphology and composition, all prepared nanofibers were subjected to different characterization techniques, such as Scanning Electron Microscopy (SEM), and Fourier Transform Infrared Spectroscopy (FTIR). In addition, its antimicrobial activity was checked both with qualitative and quantitative approaches against gram-positive *(Staphylococcus aureus)* bacteria and gram-negative *(Escherichia coli)* bacteria. The results suggest that increasing ZnO concentration kills and inhibits bacterial growth more proficiently compared to increasing aloe vera concentration in electrospun nanofibers; the highest antimicrobial was found with 4% ZnO, killing almost 100% of gram-positive *(Staphylococcus aureus)* bacteria and 99.2% of gram-negative *(Escherichia coli)* bacteria. These fabricated nanofibers have potential applications in medical devices and would help control the spread of many diseases.

## 1. Introduction

The WHO on 11 March 2020 declared the COVID-19 virus a global pandemic [1]. Currently the world is moving towards the fourth wave of COVID-19, with more than 13 million confirmed cases and 3 million deaths [2]. This pandemic has increased the demand for antimicrobial products in daily life, including medical devices [3]. In addition, a large shortage of PPEs for front line workers has been observed in this pandemic [4,5]. As nanomaterials can be applied to textiles [6] and other polymers (including biopolymers and synthetic) [7] by coating techniques or absorption, functionalized product can be used for the desired end application such as protective clothing and medical textiles. Nanomaterials, especially nanoparticles and nanofibers, have characteristic physical and chemical properties, making them suitable for fighting against contagious diseases, by reducing their spread (medical devices) and also in their further treatments (drug delivery).

Keeping in view the waves and wide spread of viruses and bacteria, nanomembranes with special characteristics can be used in medical devices [8]. There are many techniques to develop nanofiber membranes that include drawing [9], phase separation, template synthesis [10], self-assembly, centrifugal spinning [11] and electrospinning [12]. Much research has been done on the formation of antimicrobial nanofibers; some have inherent antimicrobial activity, and some have antimicrobial properties by incorporating materials such as drugs, herbs, and semiconductors. Fouda et al. [13] found that CMCTS-PEO-AgNPs have twice the strength against microbes compared to fibers containing only silver nanoparticles. The antimicrobial behavior is due to the presence of silver and chitosan in nanofibers. Shalumon et al. [14] found a great antimicrobial activity of sodium alginate (SA)/poly (vinyl alcohol) (PVA)/ZnO against *S. aureus* and *E. coli*. Hwang et al. [15] developed the PMMA/ZnO/TiO_2_ nanomembranes and found that the PMMA/ZnO/TiO_2_ nanomembranes show higher antimicrobial activity (approx. 86.7% inhibition efficiency) under UV irradiation compared to PMMA/ZnO nanofibers (68.3%) or PMMA/TiO_2_ nanofibers (56.2%). Jing et al. [16] formed chitosan/polyethylene oxide/silver NPs nanomembranes and discovered that these composite nanomembranes have greater antimicrobial properties as compared to other membranes that do not contain silver.

Among all organic natural materials, aloe vera is one of the natural plants that has antimicrobial and wound healing properties. In addition, due to its soft feel, it is used in many of cosmetics [17]. Researchers have discovered the composition of aloe vera and found that glucomannan and acemannan are active antimicrobial agents [18]. Several studies have been conducted to use aloe vera for tissue engineering scaffolds [19]. Studies proved that aloe vera has more antimicrobial activity against gram-positive bacteria rather than gram-negative bacteria [20]. Suganya et al. [21] developed nanofibrous scaffolds constituting aloe vera/PCL. They found more strength, thinner fiber diameter, and more hydrophilic properties with aloe vera in PCL. Fatemah et al. [22] formed aloe vera/PVA nanofibers and found a 60% release of aloe vera in the first hour and approximately 90% release in 2–4 h, in phosphate buffer solution. Ibrahim et al. [23] optimized parameters for aloe vera/chitosan nanofibers and found that 90% concentration of acetic acid, a 10 cm distance from the nozzle to the collector, and 0.3 mL/h of federate were the best for fiber formation.

Along with nanofibers, much research has also been carried out with semiconductors to achieve enhanced antimicrobial and UV-resistant properties in nanomembranes. ZnO nanoparticles have generated considerable attention due to their optical, magnetic, antibacterial, UV-resistant, self-cleaning, and semiconducting properties, and their applications have been found in cosmetics, paints, ceramics, textiles, medicines, and electronics [24]. ZnO is a II–VI semiconductor compound having a wide band gap energy of 3.37 eV and large exciting binding energy at room temperature of 60 meV [25]. It also shows piezoelectricity, that is, the generation of charges upon the application of stress [26]. ZnO is UV-resistant, antimicrobial, and self-cleaning (photocatalytic behavior). All these properties are basically due to its photocatalytic behavior, which is due to the emission of reactive oxygen substances in the presence of UV light. When ZnO is illuminated with a light of energy more or equal to the band gap of ZnO, a redox reaction occurs at its surface due to the generation of electron hole pairs. The photocatalytic reaction gives rise to reactive oxygen species (ROS), which include hydroxyl radicals, superoxide ions, and hydrogen per oxides. Much effort has been made to study ZnO as a promising antimicrobial agent, UV-resistant agent and photocatalyst [27]. Many researchers have used ZnO nanoparticles to induce UV protection and antimicrobial properties in nanomembranes. Thakur et al. [28] developed a composite membrane of PVA and ZnO nanoparticles and found excellent antimicrobial activity against *S. aureus* and *E. coli.* Sekar et al. [29] developed PVA/iron doped ZnO nanomembranes and checked their antimicrobial activity against *S. aureus* and *E. coli*. They observed that pure PVA does not exhibit any antimicrobial behavior, whereas composite nanofibers having 4, 8 and 12 wt% Fe-ZnO NPs inhibited microbial growth of 13 ± 0.3 mm, 16 ± 0.3 mm and 19 ± 0.3 mm for *S. aureus* and 9 ± 0.4 mm, 11 ± 0.4 mm and 14 ± 0.4 mm for *E. coli*, respectively.

In our previous work [30], we developed PVA/aloe vera nanofibers and found excellent antimicrobial activity; however, to boost the antimicrobial activity and impart durable antimicrobial behavior we had to functionalize these nanofibers with materials having antimicrobial and photocatalytic properties. In our present study, we developed PVA/aloe vera/ZnO nanomembranes and used four different concentrations of aloe vera and ZnO NPs and checked their morphological structure by SEM and chemical composition by FTIR. The developed nanofibers were tested for their antimicrobial activity against gram-negative *E. coli* and gram-positive *S. aureus* bacteria.

## 2. Materials and Methods

### 2.1. Materials

ZnO nanoparticles (nano powder, particle size <100 nm) and polyvinyl alcohol (PVA) (MW: 85,000–124,000 and 87–89% hydrolyzed) were purchased from Sigma-Aldrich Corporation (St. Louis, MO, USA); aloe vera (AV) gel was extracted from the Pakistani aloe vera plant.

### 2.2. Preparation of AV/ZnO/PVA Nanofibers

The nanofiber sheets were prepared using the electrospinning technique. Four solutions were prepared with varying concentrations of AV (constant ZnO) and four different solutions were prepared with varying concentrations of ZnO (constant AV). A total of 10% by weight of PVA was dissolved in a certain amount of deionized water and stirred at 450 rpm, keeping the temperature of the solution at 60 °C for 60 min. Then, a certain amount of AV and ZnO was added according to the design of the experiment, as shown in Table 1.

**Figure 1:**
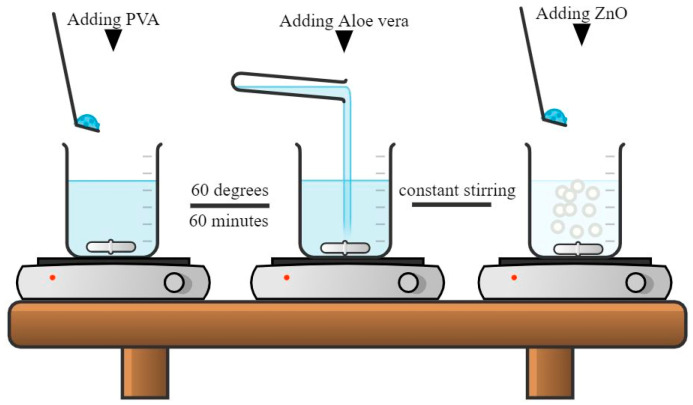
Aloe vera/PVA/ZnO solution formation process for electrospinning.

After 60 min, a homogeneous solution of AV/ZnO/PVA according to the DOE was obtained and the solutions were ready for electrospinning. Figure 1 depicts the solution formation process for the electrospinning machine.

**Figure 2:**
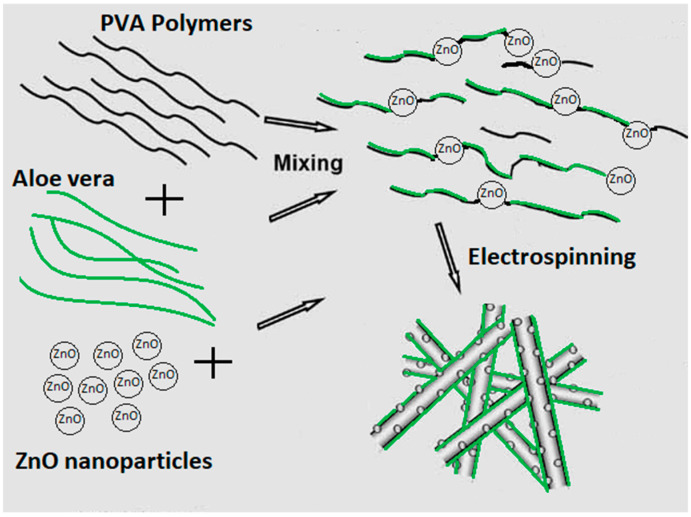
Incorporation of ZnO and aloe vera in nanofibers.

To produce AV/ZnO/PVA electrospun nanofibers, a syringe was filled with the prepared polymer solution and a voltage generator was applied to the syringe, maintaining a voltage of 17 kV and a 20 cm distance between the collector and the tip of the syringe, at an ambient temperature of 25 °C and 65% relative humidity. As the voltage was applied, the AV/PVA/ZnO fibers started to generate and were collected on the collector of the electrospinning machine. ZnO NPs were embedded in nanofiber structure, and some are also embedded on the surface of PVA nanofibers; a schematic illustration of the fibers formed is presented in Figure 2. All characterizations were performed on prepared nanofibers sheets.

### 2.3. Characterization

The prepared nanofiber sheets were subjected to different types of characterization techniques to check the effect of the variables (from Table 1) on the surface morphology, chemical composition and antimicrobial behavior of the electrospun nanofibers. To check the surface morphology of the electrospun nanofibers, we analyzed the nanofibers using a scanning electron microscope (SEM) Hitachi model S-3400N scanning electron microscope (SEM) from the Lithuanian Energy Institute. The average diameter for specimens spun from different AV or ZnO concentrations was calculated from 100 measurements each. To test the chemical interactions, the analysis of the prepared electrospun nanofibers were studied by using an FTIR spectrophotometer (Perkin Elmer; Buckinghamshire, UK).

### 2.4. Antimicrobial Activity of Nanofibers

#### 2.4.1. Qualitative Analysis

The antimicrobial activity of all the developed samples was analyzed using the agar diffusion test (qualitative method). For this purpose, the ISO 20645:2004 standard method was followed. The AV/ZnO/PVA electrospun nanofibers were tested against gram-negative bacteria (*E. coli*) and gram-positive bacteria (*S. aureus*) following the test standard ISO 20645:2004. A total of 10 mL of prepared agar was poured into a sterilized petri dish and left to congeal. For the upper layer of the petri dish, 150 mL of agar were inoculated with a bacterial working culture of 1–5 × 10^8^ cfu/mL. To have an even distribution of bacteria, the vessel was placed on an orbital shaker. A total of 5 mL of this inoculated suspension was poured as the top layer on petri dishes and then the agar was left to congeal. Electrospun nanofiber specimens were placed on agar plates with sterilized tweezers and the plates were then incubated for 24 h at 37 °C. The growth was examined, and the inhibition zone was calculated from the middle of the sample to the edge of inhibition zone. The inhibition zone was measured in millimeters from different possible directions and a mean was calculated for each sample.

#### 2.4.2. Quantitative Analysis

To check the antimicrobial activity of semiconductors like ZnO, we performed the quantitative analysis following ISO 20743:2013. The study was performed against gram-positive *S. aureus* and gram-negative *E. coli* by the quantitative measurement using the plate count method, and the results were expressed by a bacterial reduction percentage (R%). For this test, the number of viable species in suspension was estimated and the percentage reduction was measured based on colonies from the untreated sample.

## 3. Results and Discussion

**Figure 3:**
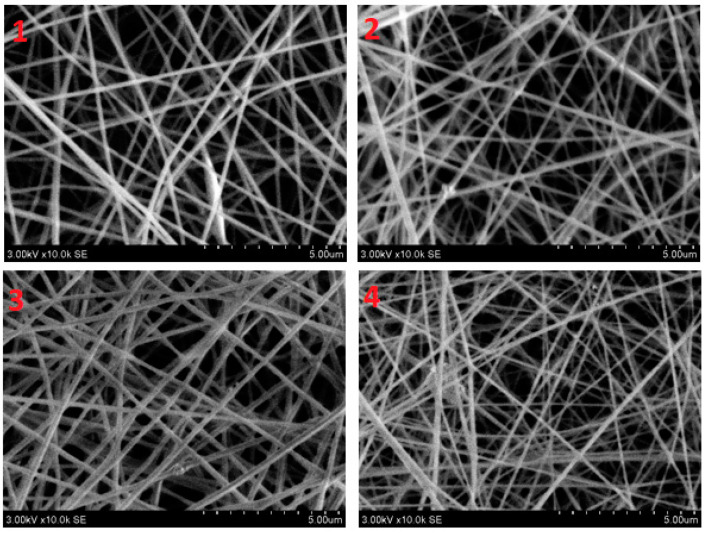
SEM images of electrospun nanofibers: “1”—10% PVA/1% aloe vera/0.5% ZnO NPS; “2”—10% PVA/2% aloe vera/0.5% ZnO NPS; “3”—10% PVA/3% aloe vera/0.5% ZnO NPS; and “4”—10% PVA/4% aloe vera/0.5% ZnO NPS.

**Figure 4:**
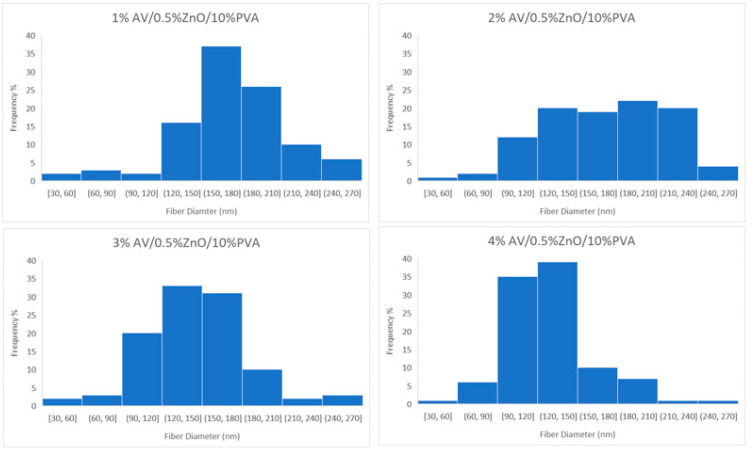
Diameter distribution of developed nanofibers in samples 1, 2, 3 and 4 with 1%, 2%, 3% and 4% aloe vera, respectively, and containing constant amount of ZnO and PVA.

The SEM examination concentrated on the morphology of nanofibers. Figure 3 shows that the nanofibers had a smooth and uniform surface with very little agglomeration due to the presence of ZnO nanoparticles in them. To measure the mean diameter of nanofibers in a nanomembrane, 100 readings were taken for each sample from different fibers and from different locations of the same fiber, and then the average diameter of nanofibers was calculated. The histograms in Figure 4 show the number of fibers per diameter range. In nanomembrane sample 1, with 1% AV, a 180 nm mean diameter of nanofibers was obtained. In sample 2, where the AV concentration was increased to 2% with a constant ratio of ZnO and PVA, a mean diameter of 176 nm of nanofibers was observed. Sample 3 showed a 148 nm mean diameter of nanofibers of a nanomembrane containing 3%, while sample 4 showed a mean diameter of 130 nm for a nanofiber membrane containing 4% AV. In addition, it can be observed from Figure 4 that sample 4 had the highest number of fibers in the 90–120 nm range and the lowest number of nanofibers in the 210–270 nm range, compared to all other prepared samples. From the above data, a tendency to decrease in diameter with an increase of AV concentration can be observed. This can be explained by the fact that the electrostatic forces and columbic forces increases when the concertation of AV was increased while keeping the concentration of ZnO and PVA constant. To test whether the decrease in the diameter of the nanofibers by increasing AV concentration was significant, *t_α_* was calculated (*t_α_ =* 68.26). The calculated *t_α_* value is much higher than the statistical value with a reliability of *α* = 0.95 (*t_αst_ =* 1.98), and this clearly demonstrates that the difference between the average diameters of the nanofibers spun at different AV concentrations in the electrospinning solution is significant.

**Figure 5:**
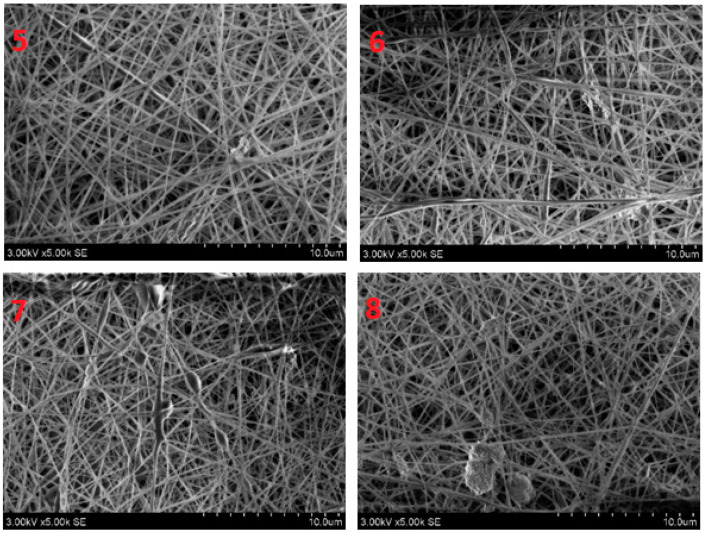
SEM Images of electrospun nanofibers: “5”—10% PVA/0.5% aloe vera/1% ZnO NPs; “6”—10% PVA/0.5% aloe vera/2% ZnO NPs; “7”—10% PVA/0.5% aloe vera/3% ZnO NPs; “8”—10% PVA/0.5% aloe vera/4% ZnO NPs.

**Figure 6:**
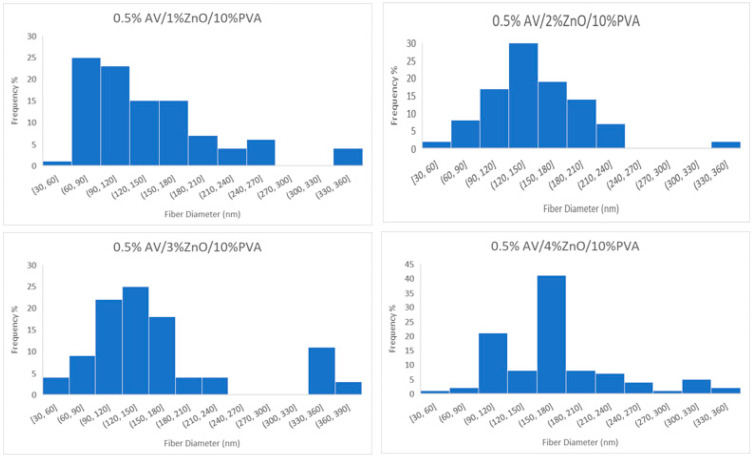
Diameter Distribution of electrospun nanofiber samples 5, 6, 7 and 8 containing 1%, 2%, 3% and 4% ZnO NPs, respectively, with constant amounts of aloe vera and PVA.

Figure 5 shows the nanofiber mats electrospun with an increasing concentration of ZnO nanoparticles while keeping the AV content at 0.5%. The electrospun fibers were relatively smooth, regular and straight, like pure PVA nanofibers. As the concentration of ZnO increased from 1% to 4%, bead-like structures appeared on the surface of the nanofiber mats. The beads were more prominent at higher concentrations of ZnO nanoparticles, as the nanoparticles agglomerate with polymer solutions in higher concentrations. The point to note is that the rough beads shows the presence of ZnO nanoparticles while the smooth beads shows the presence of only thick polymer. The average fiber diameter did not change visibly from 1% ZnO to 4% ZnO. Only some minor changes in diameter were observed with an increasing ratio of ZnO nanoparticles, such as 145 nm for 1% ZnO, 149 nm for 2% ZnO, 155 nm for 3% ZnO and 165 nm for 4% ZnO nanoparticles. However, it can be observed from Figure 6 that the peak of diameter distribution slightly moves to the right with an increase in the concentration of ZnO nanoparticles in the electrospinning solution. Furthermore, the calculated *t_α_* (*t_α_ =* 25.27) is higher than the statistical one (*t_αst_ =* 1.98 with reliability *α* = 0.95). This confirms that the difference between average diameters of nanofibers spun from different concentrations of ZnO in the electrospinning solution is significant.

### 3.1. FTIR

**Figure 7:**
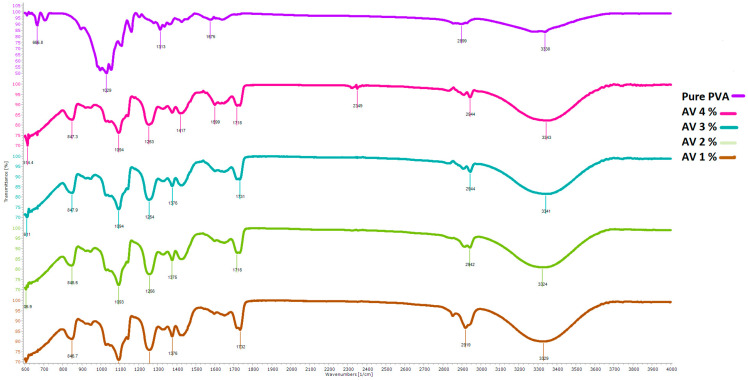
FTIR analysis of Samples 1, 2, 3 and 4 with 1%, 2%, 3% and 4% aloe vera contents, respectively.

For the chemical examination and to check the functional groups attached in the nanofibers, Figure 7 shows the results of FTIR of nanofibers containing 1%, 2%, 3% and 4% AV, ZnO (0.5%) and 10% PVA. The x-axis shows the wave number, and the y-axis shows the transmission percentage. Pure PVA nanofibers have different fingerprint regions (600 to 1400 cm^−1^) as compared to all other nanofibers, and the characteristic -OH peak is less broad than for all samples formed. The reason can be associated with the presence of AV present in all other samples, which has a higher quantity of -OH as compared to pure PVA. For the remaining blends of AV/ZnO/PVA, the fingerprint region is almost the same, only having some differences in characteristic peaks that correspond to -OH. The characteristic -OH increases with the increase in AV concentration from 1% to 4%, depicting more hydroxyl groups with increasing concentration. There seems to be no new peak in all blended samples, and this shows that there is no chemical reaction between aloe vera, ZnO, and PVA.

**Figure 8:**
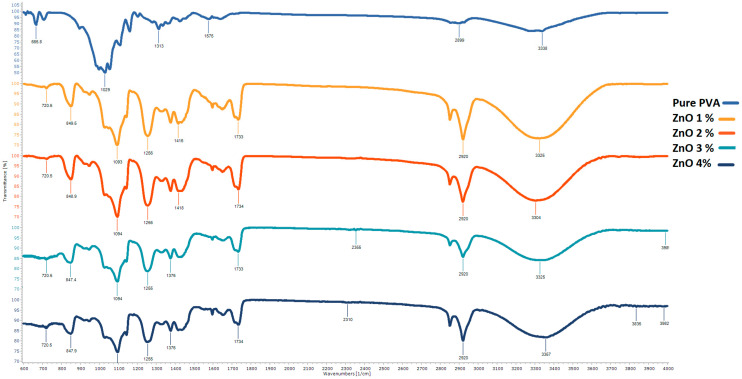
FTIR analysis of Samples 5, 6, 7 and 8 with 1%, 2%, 3% and 4% ZnO NPs, respectively.

The FTIR spectra of the nanofibers with variation in ZnO nanoparticles are shown in Figure 8 below. The fingerprint region (600 to 1400 cm^−1^) of PVA is different from all other AV/ZnO/PVA composite nanofibers. Additionally, the -OH peak in pure PVA is less broad compared to other composite nanofibers, depicting a smaller quantity of hydroxyl groups in comparison. In addition, with the increase in ZnO concentration, there is no prominent difference in the characteristic -OH peaks of all composite nanofibers. All the FTIR spectra of composite fibers are almost identical, showing no new bonds between AV, ZnO and PVA.

### 3.2. Antimicrobial Activity of AV/PVA/ZnO Nanofibers

The antimicrobial activity (qualitative analysis) of all samples of AV/PVA/ZnO nanofibers with variations in AV or ZnO concentration was tested according to EN ISO 20645:2004. It was observed that all the prepared samples shrunk when they were placed on the cultured agar plates; the reason behind this is that the PVA with AV becomes so hydrophilic that upon contact with mild wet surfaces it tends to shrink. The antimicrobial activity of AV is due to the presence of anthraquinones, etc., present in their gel structure. Another constituent of AV gel is cinnamic acid, which inhibits glucose uptake by resting bacteria, thus inhibiting the growth of bacteria. ZnO is a semiconductor that exhibits antimicrobial, UV-resistant, and photocatalytic properties due to its low band gap of 3.31 eV. When an incident ray equal to or greater than the band gap between the valance band and the conduction band is absorbed by the valance band electron (e^−^), it results in the excitation of the valance band electron. In this excitation state the electron from the valance shell will jump to the conduction shell, just because it has absorbed the incident ray and has sufficient energy to leave its orbit and jump to higher energy orbits. This movement of the electron from the valance band to the conduction band results in an electron hole (electron deficiency) in the valance band (h^+^). This deficiency in the valance band has a highly localized electron vacancy in the ZnO NPs. Both e^−^ and h^+^ participate in the redox reactions with the microorganisms absorbed on the surface of the ZnO NPs, thus disrupting their cell wall and killing the bacteria. Results of antimicrobial activity of all prepared samples against *S. Aureus* are presented in Table 2. It is observed that as the concentration of AV increased, the zone of inhibition also increased. Sample 1 showed an average zone of inhibition of 8.5 mm, sample 2 showed 10 mm, sample 3 showed 12 mm, and sample 4 showed 15 mm, as measured from the center of samples.

Samples 5, 6, 7 and 8 had very small zones of inhibition. Even with the increasing concentration of ZnO from 1% for sample 5 to 4% for sample 8, there appeared to be no increase in the zone of inhibition by nanomembranes. This can be explained by the fact that the ZnO NPs are embedded within the nanofiber structure and would kill only the bacteria contacting the fiber surface. Contrary to ZnO, aloe vera is hydrophilic, and when it is released from the nanofiber into the agar solution, it kills the bacteria, and a clear zone of inhibition is seen with an increasing amount of AV, as shown in Table 2.

Quantitative analysis of all electrospun nanofibers was performed to check their level of antimicrobial performance following the standard ISO 20743:2013. The antimicrobial activity of all prepared electrospun nanofibers was checked against gram-positive and gram-negative bacteria, and the results are presented in Table 2.

**Figure 9:**
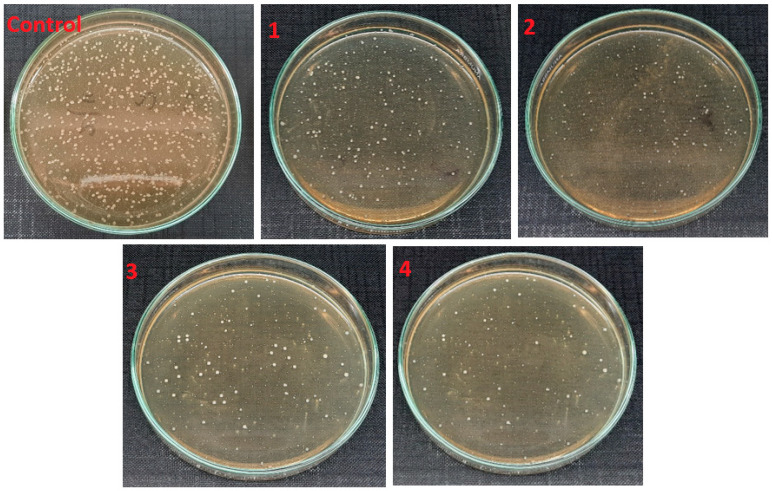
Antimicrobial activity of electrospun nanofibers samples 1, 2, 3 and 4 against *S. Aureus* with comparison to control (with no sample).

**Figure 10:**
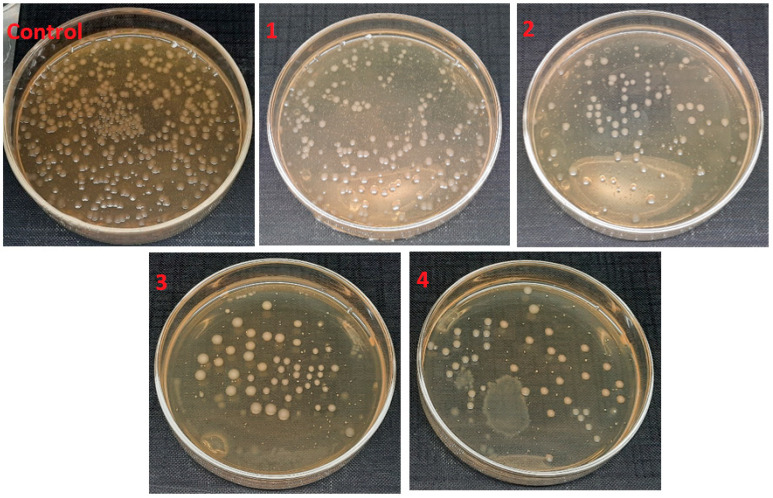
Antimicrobial activity of electrospun nanofibers samples 1, 2, 3 and 4 against *E. Coli* with comparison to control (with no sample).

Figure 9 shows the antimicrobial activity of electrospun nanofibers with variation in aloe vera against gram-positive bacteria and their percentage of bacterial reduction, as shown in Table 3. As compared to the control test (without sample), a decrease in the bacterial colonies forming unit (cfu) was observed with an increase in the amount of AV from 1% to 4%, where 1% of AV killed almost 75.5% *S. Aureus* bacteria, 2% of AV killed 79.1% bacteria, 3% killed 86.1% bacteria and 4% of AV showed the highest antimicrobial activity results, killing almost 91.2% *S. Aureus* bacteria in the suspension. A similar behavior was observed against *E. coli* as shown in Figure 10. An increase in the percentage of aloe vera decreased the *E. coli* by up to 86.9%. Compared to antimicrobial activity against *S. aureus*, the antimicrobial activity against *E. coli* was quite low. This can be explained by the fact that the gram-negative bacteria *E. coli* has a thicker cell wall as compared to the gram-positive bacteria *S. aureus*, where the thicker wall resists the constituents of AV more, and hence the activity of AV is less effective against *E. Coli*.

**Figure 11:**
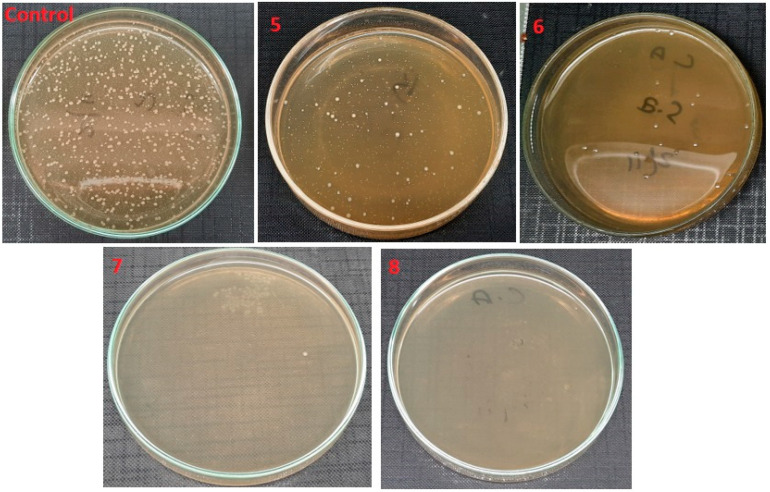
Antimicrobial activity of electrospun nanofibers samples 5, 6, 7 and 8 against *S. Aureus* with comparison to the control (with no sample).

**Figure 12:**
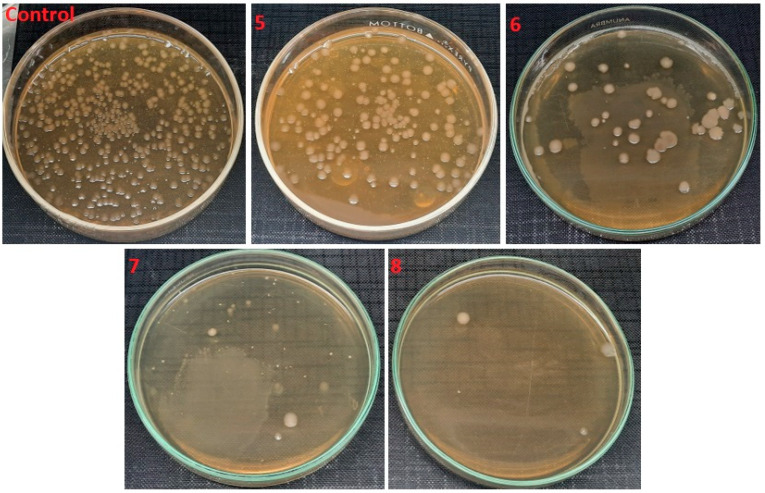
Antimicrobial activity of electrospun nanofibers samples 5, 6, 7 and 8 against *E. Coli* and compared to the control (with no sample).

Figure 11 shows the antimicrobial activity of electrospun nanofibers with variation in ZnO NPs concentration against gram-positive bacteria, and their percentage of bacterial reduction is shown in Table 3. When antimicrobial activity is compared with the control test, a decrease in bacterial cfu was observed with the increasing amount of ZnO NPs from 1% to 4%: 1% ZnO NPs killed almost 93.5% of *S. Aureus* bacteria, 2% of ZnO NPs killed 97.2% of bacteria, 3 % of ZnO nanoparticles killed 99.8% of bacteria and 4% showed exceptional results, killing almost all of the bacteria in the suspension. Similar behavior was observed against *E. coli*, as shown in Figure 12. An increasing percentage of ZnO NPs killed *E. coli* bacteria by up to 99.2%. As compared to antimicrobial activity against *S. aureus*, the antimicrobial activity against *E. coli* was lower. This can be explained by the fact that the gram-negative bacteria *E. coli* has its cell wall sandwiched between two phospholipid bilayers compared to the gram-positive bacteria *S. aureus* that have no outer phospholipid bilayer to protect their cell wall, and therefore the outer lipid layers resist the e^−^ and h^+^ participating in the redox reactions at the surface of ZnO NPs more, and less *E. coli* are killed by this activity.

## 4. Conclusions

Electrospun nanofibers were made with varying concentrations of aloe vera (1%, 2%, 3%, 4%) keeping constant concentrations of ZnO NPs (0.5%), and with varying concentrations of ZnO NPs (1%, 2%, 3%, 4%) with constant concentration of aloe vera (0.5%). SEM analysis showed a smooth surface of nanofibers with some agglomeration with the increase in percentage of ZnO NPs. FTIR analysis showed no additional functional groups, showing no chemical reaction between PVA, aloe vera and ZnO nanoparticles. Qualitative antimicrobial analysis showed that samples containing different concentrations of ZnO NPs did not exhibit any zone of inhibition, due to the lack of mobility of ZnO NPs from fibers to the external medium. Quantitative antimicrobial analysis showed excellent results with samples having different concentrations of ZnO NPs as compared to samples having variation in aloe vera. The reason behind this can be explained by the fact that aloe vera is consumed while killing the bacteria, while ZnO NPs remain embedded into the nanofiber structure and show everlasting antimicrobial activity. Additionally, ZnO NPs are semiconductors, and their redox reactions kill bacteria more efficiently as compared to natural aloe vera, which does not exhibit this property. The prepared AV/PVA/ZnO NPs nanomembranes have potential application in medical devices due to their excellent antimicrobial properties.

## Figures and Tables

**Table 1:** Design of experiment for the formation of aloe vera/PVA/ZnO nanomembranes.

Sample Number	Polyvinyl Alcohol	Aloe Vera	Zinc Oxide Nanoparticles
1	10%	1%	0.5%
2	10%	2%	0.5%
3	10%	3%	0.5%
4	10%	4%	0.5%
5	10%	0.5%	1%
6	10%	0.5%	2%
7	10%	0.5%	3%
8	10%	0.5%	4%
9	10%	0	0

**Table 2:** Qualitative analysis—zone of inhibition of all electrospun samples against *S. Aureus*.

Samples	1	2	3	4	5	6	7	8	9
Zone of inhibition	8.5 mm	10 mm	12 mm	15 mm	<1 mm	<1 mm	<1 mm	<1 mm	0

**Table 3:** Antimicrobial activity of all electrospun samples with variation in aloe vera and Zno NPs against *S. Aureus* and *E. Coli*.

S. No	Percentage of Bacterial Reduction After 24 h	
	*S. Aureus*	*E. Coli*
1	75.5%	60.9%
2	79.1%	73.9%
3	86.1%	81.8%
4	91.2%	86.9%
5	93.50%	83.14%
6	97.20%	92.96%
7	99.80%	96.87%
8	100%	99.20%

## Data Availability

Not applicable to this study.
